# Reliable evaluation for the AI-enabled intrusion detection system from data perspective

**DOI:** 10.1371/journal.pone.0334157

**Published:** 2025-10-31

**Authors:** Hui-Juan Zhang, Kai Yang, Peng Ran, Shen He, Jia Chen

**Affiliations:** Research Institute of Safety Technology, Research Institute of China Mobile, Beijing, China; Beijing Institute of Technology, CHINA

## Abstract

As the primary link in cybersecurity, the intrusion detection system (IDS) is of indispensable importance. Many studies have proposed sophisticated artificial intelligence (AI) models to detect intrusion behavior from a large amount of data, yet they have ignored the fact that poor data quality has a direct impact on the performance of IDS. The poor data quality is mainly attributed to the interference and damage, such as data tampering, poisoning, and corruption, which leads to decision-making deviations, triggering a serious trust crisis of model application. This paper proposes a multi-indicator comprehensive evaluation method (MICEM) to ensure the reliability of AI decision-making from data perspective. First, several evaluation indicators are established to analyze the potential risks that intrusion detection data may face from the different dimensions, and specific quantitative methods are provided. Second, a comprehensive evaluation is conducted based on the results of each indicator to determine the quality of the intrusion detection data as a whole, thus guaranteeing the usability and reliability of AI-enabled IDS. Finally, the effectiveness and practicality of the proposed MICEM are fully verified by evaluating the benchmark-CICIDS2017 dataset and the real intrusion detection dataset.

## I. Introduction

In the digital era, the intrusion detection system (IDS), is the first line of defence in maintaining the security and stability of the network environment [1–2]. Intrusive behaviour poses a threat to the network, including unauthorised access, malware attacks and denial-of-service attacks [3]. Most intrusion detection methods in traditional mode use the preset rules and statistical models as their infrastructure, and identify intrusion behaviors through feature extraction and behavior matching of known attack patterns [4–6]. However, with the continuous evolution of network attack methods, traditional security protection measures are struggling to cope with the increasingly complex threats.

In the field of network security, the rapid development of machine learning technology, especially the continuous innovation of deep learning algorithms, has made their application in intrusion detection a research focus [7–8]. Machine learning technology, with its powerful reasoning capabilities, is able to analyse large amounts of network data to automatically identify potential abnormal behavioural features and patterns [9]. This enables effective detection and early warning of unknown network intrusion behaviours and provides intelligent, efficient solutions for network security protection [9]. Over the past decades, numerous investigators have concentrated their efforts on designing models, such as optimizing their structures or enhancing their learning algorithms to enhance the reliability of IDS. For instance, Zhang et al. proposed a detection method based on an improved stacked sparse autoencoder (ISSAE) [10]. This method simplifies and sparsifies the basic units of the sparse autoencoder (SAE), combining basic features with higher-level abstract expressions to solve the issue of missing network traffic data. A dual-stage deep learning model, is studied to address class imbalances in intrusion detection data [11]. In this model, the SAE detects anomalies and extracts features. A layered deep learning model combining convolutional neural network (CNN) and bidirectional long short-term memory (Bi-LSTM) architectures is then employed for multiclass classification. Moreover, Ho et al. designed an IDS based on a modified convolutional neural network (MCNN) that classifies all network traffic packets as either benign or malicious, thereby enhancing the internet security [12]. Dutt et al. designed an immune intrusion detection system (IIDS) that mimics the natural immune system [13]. In this IIDS, statistical modeling-based anomaly detection (SMAD) serves as an interface to the innate immune system (IIS), capturing the initial network traffic and identifying first-hand vulnerabilities. Then, the immune system is activated based on neural network algorithms, to improve the effectiveness of intrusion detection by capturing relevant features from the header and payload sections. Other intrusion identification detection model can be found in [14–16]. Although AI-based network intrusion detection methods have improved in terms of accuracy and efficiency, some challenges and problems remain. Firstly, the effective training of intrusion detection models is highly dependent on massive, high-quality and reliable network data [17]. However, current network data is generally characterized by issues such as sample imbalance and incompleteness, which significantly restrict the model’s generalization ability. Furthermore, during storage, transmission and use, data is vulnerable to various forms of interference and damage risks [18], such as data tampering, poisoning, deletion or corruption. This can lead to serious deviations in the model’s prediction and decision-making processes, potentially causing major network security incidents. Moreover, similar security issues also exist at the lower layers of communication network models, such as the physical layer. For instance, Kang et al. proposed a novel recursive-iterative approximation to explore the fundamental limits of covert communication concealed by randomly activated overt users [19].

In fact, breakthroughs in the development of AI have been made possible by the development of data [20–21]. In particular, as the emphasis transitions from model optimizing to data improvement, data scientists often spend nearly twice the time on data loading, cleansing and visualization in comparison to model training, selection and deployment [22]. “Garbage in, garbage out,” more and more researchers are realizing the impact of data on the reliability of IDS [23]. A multi-level hybrid support vector machine and extreme learning machine based on modified k-means (SVM-ELM-MK), is proposed [24]. In this well-designed algorithm, a modified *k*-means is used to build new small training datasets representing the entire original training dataset, which can reduce the training time of SVM-ELM, and improve the performance of intrusion detection system. Ayubkhan et al. have developed a denoising autoencoder combined with a light gradient boosting machine (DA-LGBM) classifier for mitigating the effects of noise and corrupted data, to ensure the stability of the IDS [25]. In this DA-LGBM, the DA is capable of removing noise and corruptions in the network traffic data, to enhance the features learning capacity required for classification, thereby avoiding the deviations. Furthermore, to effectively solve the data imbalance problem, Park et al. improve the conventional GAN to generate credible synthetic data for small attack traffic [26]. Subsequently, the stack autoencoders are trained to improve the stability of IDS. Lee et al. address the data imbalance problem by generating new virtual data similar to the existing data through Generative Adversarial Network (GAN) models [27]. Then, based on the hybrid GAN-generated dataset, a random forest model is trained to improve the detection performance. However, the methods outlined in [24–27] may be limited in their capacity only focusing on one aspect of the data and ignore other crucial indicators. This may hinder their ability to provide a comprehensive assessment of the reliability of the decision-making outcomes of AI-based IDS.

In light of the aforementioned analysis, this paper puts forward a multi-indicator comprehensive evaluation method (MICEM) for evaluating the reliability of AI-enabled IDS data. Firstly, multiple assessment indicators are established to thoroughly analyse the potential risks that intrusion detection data may face, and meanwhile specific quantitative methods are given respectively. Secondly, a comprehensive evaluation is conducted based on the assessment results of each indicator to determine the quality of the entire intrusion detection data set, thus guaranteeing the usability and reliability of AI-Enabled IDS. Finally, by evaluating the intrusion detection data in benchmark dataset and a real application scenarios, the effectiveness and practicality of the proposed method are fully verified. It is worth noting that, the proposed MICEM has a high degree of scalability in terms of evaluation indicators in different application scenarios.

The outline of this paper is organized as follows. Section II introduces the proposed MICEM, consisting of analysis and quantization of evaluation indicators and the comprehensive evaluation method in detail. Subsequently, the application process of MICEM is shown in detail in benchmark-CICIDS2017 dataset and a real-world in intrusion detection scenarios in Section III. Moreover, the experimental results and the comparisons are also discussed to demonstrate the effectiveness of the proposed MICEM in Section III. The conclusions are given in Sections IV.

## II. Multi-indicator comprehensive evaluation method

**Fig 1:**
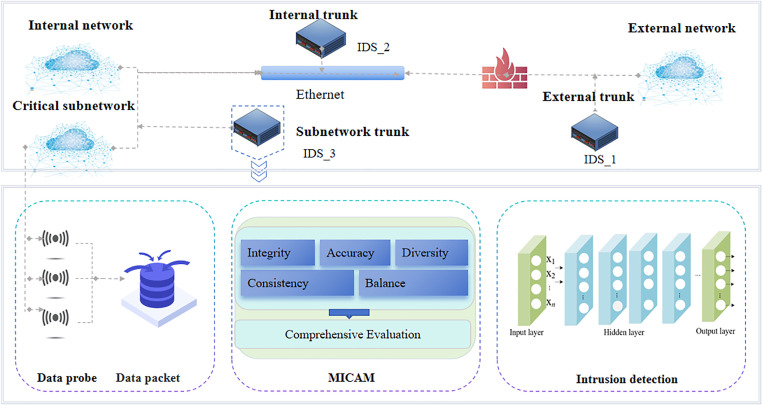
The framework of the proposed MICAM.

In this section, a MICEM is proposed for the evaluation of intrusion detection data. As demonstrated in Fig 1, IDS can be deployed on the external network, internal network and critical subnetwork. For example, IDS_1 is applied inside the firewall to monitor traffic that penetrates the firewall and detect covert attacks that bypass the firewall (such as tunnel encrypted traffic). IDS_2 and IDS_3 are arranged into internal trunk and subnetwork trunk respectively to detect the normal behaviour, with the purpose of facilitating the real-time monitoring of network traffic and activities. Taking IDS_3 as an example, the data such as system logs, network traffic, file change records and user activity status are captured and packaged into data packets through data probes deployed in different network nodes. In the subsequent phase of the process, multiple indicators, consisting of completeness, accuracy, consistency, diversity and balance, are meticulously designed to evaluate the data condition separately by calculating individual score of each indicator. Then, a comprehensive evaluation method, integrating the evaluation results of above indicators, is developed to provide a synthetically evaluation for the data quality. This can provide a powerful basis for subsequent data analysis. Finally, the evaluated data is fed into an analyzer (neural network) and analyzed to identify potential attacks. In the following section, the MICEM will be introduced in detail.

### A. Design and calculation of pivotal indicators for data reliability evaluation

**1) Integrity:** Data integrity means that the data is not lost, damaged or tampered during the processes of collection, storage, transmission and processing [28]. Data integrity is key to the accuracy and decision reliability of AI models. However, missing data is a common problem in the actual operation of AI models. This can be caused by a variety of factors, such as differences in heterogeneous network environments, unstable network signals, or transmission link failures, all of which may result in data collection failures as the data collection device is unable to successfully transmit the data to the intended receiving point. In particular, attackers adopt DDOS-like attacks in an attempt to put IDS into an overloaded operating state, causing significant delays or even complete interruption of data collection, making it difficult to obtain and update critical information in the network in a timely manner, and bringing great challenges to the analysis work of the AI analysis model in the subsequent IDS. When AI models rely only on these incomplete data for training and real-time reasoning, decisions of AI may be faulty due to unstable features or patterns learned by the models, which can lead to serious security incidents. Besides, data accumulation is the basis for building effective detection models. However, its characteristic of large time span creates opportunities for attackers to exploit. Attackers may destroy the integrity of the data by injecting fake data or malicious samples into the data, which greatly reduces the reliability and effectiveness of the intrusion detection system, and even fails to provide due protection for network security. Therefore, the integrity of data need to evaluated, including null value detection and data mutation detection.

Null value assessment focuses on the distribution of data with null values and can be given as


$$S_{Nu}=\frac{g-b}{g}*100\%,$$ (1)

where *b* signifies the number of training data samples with null values and *g* indicates the size of data.

The injected false data usually appears random, while the real data typically conforms to a certain pattern of change. When false data is added to the original dataset, it will introduce a certain degree of variability to the data. Therefore, for data mutation detection, the rate of change of the root mean square of the data can be calculated. The degree of data variability determined in this way serves as one of the bases for judging whether the training data has been attacked by data injection


$$S_{Fa}=\left\{ \begin{array}{c} @l0,\left|c-c^{\prime }\right|\geq c\\ (1-\frac{\left|c-c^{\prime }\right|}{c})*100\%,\left|c-c^{\prime }\right|<c \end{array}\right.,$$ (2)

where *c*′ denotes the root mean square of the current sample data, and *c* denotes the average of the root mean squares of multiple historical sample data of the same batch. When the rate of change of the root mean square is too large, it indicates that there may be data mutations. It is worth noting that the generation of noise can also cause the rate of change of the root mean square to be too large, so further confirmation is needed when the data shows high mutability.

Therefore, the integrity of the intrusion detection dataset is given as


$$S_{I}=\omega_{Nu}S_{Nu}+\omega_{Fa}S_{Fa},$$ (3)

where ω_*Nu*_ and ω_*Fa*_ are the connection weight.

**2) Accuracy:** Data accuracy can measure the closeness of the collected data to the actual or expected value [29]. In intrusion detection data, when the pivotal features are erroneous or mislabeled, the model learns the wrong decision-making information and thus fails to recognize the correct pattern. This not only poses a serious threat to cybersecurity, but also greatly reduces the credibility of AI. Therefore, there is a need to detect anomalies in intrusion detection data so as to assess its accuracy. There are many methods for data anomaly detection, and the following Table 1 lists some of the commonly used methods. Different methods have different advantages and disadvantages, and in practical scenarios, it is necessary to consider the characteristics of the data, the application scenario, the required accuracy, and computational resources to choose different detection methods.

**Table 1:** The methods for data anomaly detection.

Type	Detection method	Description
Statistics	3Sigma	Outliers are values that are more than 3 standard deviations away from the mean
	Z-Score	After normalizing the data, those points whose Z-Score is above a certain threshold in absolute value are outliers
	Boxplot	Outliers are identified by plotting five-digit generalizations of the data (minimum, first quartile, median, third quartile, maximum)
	Grubbs hypothesis test	If the Z-score of a suspected point is greater than the Grubbs critical value, the suspected point is an outlier
	SOS	Stochastic Outlier Selection (SOS) identifies outliers by random sampling and statistical tests
Machine learning	K Nearest Neighbor	The distance between each point and its nearest neighbor is calculated, and then the threshold of the distance is used to determine whether it is an anomaly
	Local outlier Factor	Outliers are identified by calculating the local density deviation at each point
	Isolation Forster	Outliers are detected by building multiple isolation trees.
	One-Class SVM	Support vector machines (SVM) are used to learn the normal pattern of the data, and then points that are significantly different from the normal pattern are identified as outliers.
	AutoEncoder	The autoencoder is used to learn the normal pattern of the data, and then the reconstruction error is calculated to identify the outliers.
Clustering algorithm	DBSCAN	Density-Based Spatial Clustering of Applications with Noise (DBSCAN) clusters data points according to their density, and isolated points that are not contained by effective clustering are treated as outliers.
Other	Rule-based approach	It defines the characteristics of outliers based on business rules or domain knowledge, and then detects outliers through these rules.

After anomalous data is discovered by outlier detection technology, the accuracy of training data set can be given as


$$S_{A}=\frac{g-d}{g}*100\%,$$ (4)

where *d* is the number of training data samples with outlier.

3)** Consistency:** Data consistency is used to describe whether the same entity has the same attributes in different data sets, and whether each entity and attribute conforms to consistency constraints [30]. IDS may collect data from multiple sensors, log sources, etc., and data from different sources may have variances in format, content, and so on. Therefore, consistency evaluation allows data to maintain a uniform standard in different storage and processing links to avoid data conflicts. Inconsistency in the format of the intrusion detection data may lead to confusion for the AI model. Thus, the format of each value in the data set need be verified


$$S_{Fo}=(\frac{g-e}{g})*100\%,$$ (5)

where *e* represents the number of instances that do not match the standard format. In addition, when the content is inconsistent, such as labeling conflicting classification tags, it can cause the AI to generate false perceptions. Therefore, to verify that content data records are consistent for the same matter, content consistency should be assessed


$$S_{Co}=(\frac{g-f}{g})*100\%,$$ (6)

where *f*′ represents the number of data records with conflicting content data. The consistency of logical relationships is equally important. For the intrusion detection scenarios with the complex reasoning and identification, if the logical relationships of the data are inconsistent, the AI model may learn the wrong logic, affecting the reliability of its decisions. The consistency of relationship in data can be given as


$$S_{Lo}=(\frac{g-g^{\prime }}{g})*100\%,$$ (7)

where *g*′ represents the number of data records with conflicting sample content data between data sets.

Therefore, the consistency of the intrusion detection dataset can be computed as


$$S_{C}=\omega_{Fo}S_{Fo}+\omega_{Co}S_{Co}+\omega_{Lo}S_{Lo},$$ (8)

where ω_*Fo*_, ω_*Co*_ and ω_*Lo*_ indicate corresponding connection weights.

4)** Diversity:** Data diversity is reflected in the richness of the data sample, covering various types of network data and attack scenarios [31]. Poorly diverse samples can lead to models that are overly sensitive to specific attack patterns or features and under-recognized for others, which will further threaten the security and stability of network device. Shannon’s diversity index (SHDI) is a popular measure of diversity in community ecology. Thus, the SHDI can be adopted to evaluate the balance of intrusion detection data and can be given as


$$S_{D}=-\sum (p_{i}*\ln p_{i})*100\%,$$ (9)

where *p*_*i*_ represents the proportion of the *i*th class in the total sample and log(∙) is logarithmic function symbol. The greater the diversity of the community, the greater its uncertainty and the higher the value of SHDI.

5) **Balance:** Data balance is an important characteristic of training data. If a discrete training dataset with significant differences in the number of each class, the model tends to over-learn the features of the majority class and under-learn the features of the minority class, affecting the model’s generalization capability and leading to poor model results on real detection scenario [32]. Pielou’s Evenness Index (PEI) can be used to reflect the extent to which the number of individuals of a species is evenly distributed in a community [33]. Therefore, this PEI can also be to evaluate the balance of intrusion detection dataset and can be computed as


$$S_{B}=S_{D}/\ln(h+\text{1}),$$ (10)

where *h* indicates the total number of attack types. The closer the value of PEI is to 1, the more uniform the distribution of each category is, and the closer the value is to 0, the lower the uniformity is.

### B. Data reliability comprehensive evaluation

Based on the analysis of integrity, accuracy, consistency, diversity and balance, the comprehensive evaluation of data reliability Ω can be defined as


$$\Omega =\varpi_{I}S_{I}+\varpi_{A}S_{A}+\varpi_{C}S_{C}+\varpi_{D}S_{D}+\varpi_{B}S_{B},$$ (11)

where ***ϖ***=[*ϖ*_*I*_, *ϖ*_*A*_, *ϖ*_*C*_, *ϖ*_*D*_, *ϖ*_*B*_] is the connection weight vector. In fact, each evaluation indicator is interdependent and interact with each other, such as the integrity of data affecting the consistency of data, which in turn affects the accuracy of data. Therefore, it is necessary to comprehensively consider the weights of various evaluation indicators to make more scientific and reasonable decisions [34]. In this manuscript, the analytic hierarchy process (AHP) will be introduced due to its simple principle and easy implementation.

Construction and verification of judgment matrix: In this AHP, for *m* evaluation indicitors of data reliability, judgment matrix **C** is first constructed and the element *c*_*ij*_ of **C** are given using 1–9 scale method proposed by Saaty (shown in Table 2), using two-by-two comparison among indicators

**Table 2:** 1–9 scale method.

Scale	Interpretation
1	Equally important compared to two indicators
3	One indicator is slightly more important than the other
5	One indicator is clearly more important than the other
7	One indicator is more strongly important than the other
9	One indicator is more extremely important than the other
2,4,6,8	Median of two adjacent indicators
Inverse	*c*_*ji*_ = 1/ *c*_*ij*_


$$𝐂=\left[\begin{array}{c} c_{11},c_{12},...c_{1m}\\ c_{21},c_{22},...c_{2m}\\ \ddots \\ c_{m1},c_{m2},....c_{mm} \end{array}\right],$$

Then, a consistency testing is performed for matrix **C**


CR=CI/CIRI,\nulldelimiterspaceRI,CI=(λmax−δ)/CI=(λmax−δ)(δ−1),\nulldelimiterspace(δ−1), (12)

where CR is the consistency ratio, λmax is the absolute value of the largest eigenvalue of the matrix **C** and *τ* is the number of non-zero eigenvalues order, *RI* is the random consistency index. It can be directly obtained by Saaty and summarized in Table 3. As shown in Table 3, when the order of matrix is 4, the value of RI is 0.9. If CR < 0.1, the matrix passes the consistency verification, otherwise it does not have satisfactory consistency and the judgment matrix **C** needs to be adjusted. In the AHP process, the reasonableness of the judgment matrix is tested by calculating the CR. If CR < 0.1, the judgment matrix is considered logically autonomous. Otherwise, the judgment matrix needs to be readjusted. This test can effectively avoid the logical contradiction in subjective judgment. If the decision-maker believes that consistency A is more important than accuracy B (A > B) and accuracy is more important than completeness C (B > C), but at the same time that completeness is more important than consistency is more important than consistency (C > A). Then, there will be a circular contradiction in the judgment matrix, and the consistency test will reject the result, forcing the decision maker to revise the judgment. Therefore, through the consistency test, AHP constrains the subjective judgment within the scope of logical self-consistency and reduces the risk of confusion or inadvertence, due to confusion or inadvertence. Meanwhile, the subjective bias caused by the confused thinking or momentary negligence is reduced.

**Table 3:** The value condition of RI.

Order of matrix	Order 3	Order 4	Order 5	Order 6	Order 7	Order 8	Order 9	Order 10
*RI*	0.58	0.9	1.12	1.24	1.32	1.41	1.45	1.49

**Normalization of weight vector:** The judgment matrix **A** passes *CR* verification, then the eigenvector of the largest eigenvalue λ_max_ is the requested weight vector, and the corresponding weight of each index is derived through normalization operation.

### C. Expansion of evaluation indicators

In fact, the proposed evaluation method in this paper has a high degree of scalability in terms of evaluation indicators in different application scenarios. For example, power system relays are key components that protect power equipment and maintain stable operation of the power system [35].With the increasing scale and complexity of power systems, AI is widely used in power systems due to its powerful learning and decision-making capabilities. It can monitor and make decisions on the operating status of power systems by collecting real-time data from power systems, such as voltage, current, power and other parameters [36]. However, the effectiveness of AI is highly dependent on the quality of the input data, and any acquisition error, communication packet loss, or network delay may trigger decision bias or even chain failures. Therefore, there is an urgent need to evaluation the data reliability to meet the stringent requirements of power systems for AI decision-making. The effects of integrity, accuracy, consistency, diversity, and balance on AI models in the power system scenario are summarized in Table 4.

**Table 4:** The effects of integrity, accuracy, consistency, diversity, and balance in the power system scenario.

Evaluation indicator	Importance
Integrity	The fault information are often hidden within the correlations of data features. If the data collection is incomplete (such as sensor communication interruption, harmonic disturbances, packet loss, false data injection etc.), the AI model may fail to capture the complete fault features due to “information gaps”, resulting in missed detections or incorrect detections.
Accuracy	Relays have extremely high requirements for data accuracy. In particular, the jamming during the communication process or physical environment may generate a significant amount of data noise, causing the incorrect decisions made by the AI model.
Consistency	In power systems, relays usually work collaboratively. Data needs to be compared and analyzed across devices and over time. If the data is inconsistent, the AI model will be unable to establish stable characteristic patterns due to “logical conflicts”, resulting in a decline in its generalization ability.
Diversity	The working environment of relays is highly complex and variable. If the data only covers a single working condition data, the AI model will “overfit” to the limited scenarios and fail in new situations.
Balance	In the relay scenario, the proportion of “normal state” data is usually much higher than that of “fault state” data. If the data is unbalanced, the AI model may be biased towards “learning” and prioritize predicting the “normal state”, resulting in a low recognition rate for faults.

In addition, the latency of end-to-end air interface and return link must be stable at the millisecond or even sub millisecond level in the power system. Excessive delay will cause relay action delay and render its protection ineffective. Thus, the timeliness of data need to be evaluated


$$S_{Ti}=\left\{ \begin{array}{c} \;\;\;0,\;h\geq h^{\prime }\\ \;\;\;100\%,\;h≺h^{\prime } \end{array}\right.,$$ (13)

where *h* represents the ideal delay value and *h*ˈ is the actual latency. When *h* is greater than or equal to *h*ˈ, timeliness meets the system requirement, and vice versa. Furthermore, deterministic reliability can be measure the failure rate during data collection and transmission process and can be defined as


$$S_{DR}=(\frac{k-k^{\prime }}{k})*100\%,$$ (14)

where *k* is the number of transmission tasks, and *k*ˈ is the number of failed transmission. The other evaluation indicators such as relevance, fairness, resource consumption rate, etc., can also be used in different application scenarios to assess the reliability of data.

## III. Results and discussion

To further demonstrate the procedures and effectiveness of the proposed MICEM, we adopted a publicly benchmark dataset-CICIDS2017 to demonstrate the implementation of MICEM in detail. Meanwhile, a practical evaluation, focusing on an AI-enabled IDS scenario was also given in this section.

### A. The evaluation for CICIDS2017 dataset.

**Fig 2:**
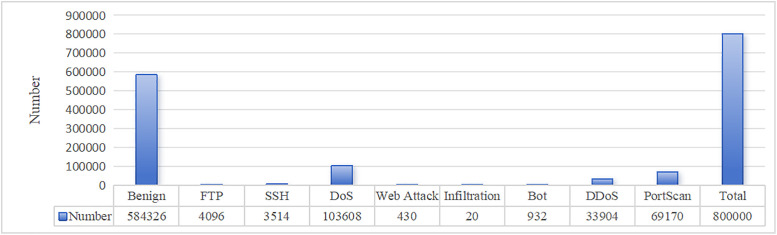
The distribution of CICIDS2017 dataset.

The CICIDS2017 dataset encompasses benign traffic and the latest prevalent attack types, more closely resembling real-world data, and can be obtained from https://www.unb.ca/cic/datasets/ids-2017.html. To conduct effective model training and validation, 800000 items were selected through random sampling to form the training set and the distribution of various types of attacks in the training set is shown in Fig 2. It can be seen that this training dateset covers 8 major attack types such as DOS attack, DDOS attacks and Web attack., and the normal samples accounted for the majority.

### A. 1 The indicators evaluation for CICIDS2017 dataset

**Fig 3:**
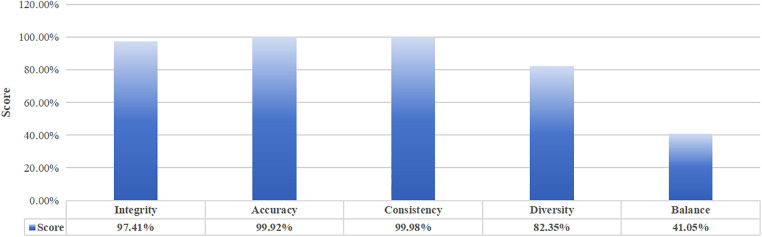
The evaluation results of indicators for CICIDS2017 dataset.

In this evaluation process, indicators: integrity, accuracy, consistency, diversity and balance are used to measure the reliability of CICIDS2017 data and the evaluation results are summarized in the Fig 3.

**Integrity** Firstly, there are 22 samples containing NaN in the whole data, so the null value detection score is 99.99%. After removing the samples containing null value, data mutation detection, by calculating the root mean square of the current sample 517.59, and calculating the average root mean square value of 10 historical samples of the same batch is 545.8, so the data mutation detection score is 94.83%. In practical applications, the weighting of each sub-indicator can be set according to the specific scenario. In order to focus on the proposed evaluation method in this paper, it is stipulated that all completeness indicators have the same weighting for each sub-completeness item. Thus, the integrity is 97.41%.

**Accuracy:** The dataset for intrusion detection is large in size, so the simple and easy-to-administer 3Sigma principle is used to detect outliers and thus evaluate the accuracy of the data. A total of 4026 samples were found to contain outliers, so the accuracy score is 99.92%.

**Consistency:** The consistency of format and content detection, 0 items were found, so the score of two items are 100%. In the consistency of relationship detection, 472 logical relationships were abnormal, so the score for this item was 99.94%. Thus, the consistency score is 99.98%.

**Diversity:** In this evaluation dataset, there are (1 normal+7 attack types), and the distribution probabilities of 8 types of label seach type are calculated separately, which are, in order 0.73, 0.005, 0.0044, 0.13, 0.00054, 0.000025, 0.0012, 0.042, 0.086. Then, Shannon entropy calculation is performed: 0.23, 0.0265, 0.024, 0.265, 0.004, 0.00026, 0.008, 0.133, 0.21 and the result of the summation is 0.8325. Therefore, the score of diversity is 90.18%.

**Balance:** Based on the result of diversity, the value of PEI is 41.05 by the Eq.10, and that means the score of balance is 41.05%.

### A. 2 Comprehensive evaluation for CICIDS2017 dataset

Based on the evaluation scores of the above indicators, the comprehensive evaluation of data reliability in this system is performed next.

Method 1: If all indicators are equally important in this scenario, i.e., the weight factor of each indicator is 0.2, thus the reliability score of intrusion detection data is:

Ω_A_=0.2*95.06%+0.2*99.92%+0.2*99.95%+0.2*90.18%+0.2*39.6%=85.71%.

Method 2: The AHP can combine the qualitative information such as the decision maker’s empirical judgment with the quantitative information such as the data, so as to make the decision-making results more scientific and reasonable. Next, the AHP will be used to calculate the weight of each evaluation index, so as to evaluate the reliability of intrusion detection data in a more reasonable and comprehensive way. First, the subjective degree of scoring based on the importance of each indicator is used to obtain a judgment matrix C


$$𝐉=\left[\begin{array}{c} 1,\;0.2,\;0.143,\;3,\;6\\ 5,\;1,\;0.33,\;7,\;9\\ 7,\;3,\;1,\;6,\;9\\ 0.33,\;0.143,\;0.167,\;1,\;2\\ 0.167,\;0.11,\;0.11,\;0.5,\;1 \end{array}\right]$$

After solving, the maximum eigenvalue is 5.29 and the number of non-zero eigenvalues is 5, which also means that the order of matrix is 5. By querying the Table 3, when the matrix order is 5, the value of RI is 1.12. Thus, consistency checking can be done next:


CR=CI/RI=(5.29−55−1)/(5.29−55−1)1.12\nulldelimiterspace1.12=0.06≺0.1,

where the judgment matrix **C** satisfies the consistency requirement. The maximum vectors corresponding to the maximum eigenvalue is [0.2131, 0.4208, 0.8757, 0.888, 0.0519]. Furthermore, after normalizing the eigenvector of the largest eigenvalue, the weight of each indicator is [0.087, 0.172, 0.358, 0.363, 0.02]. Hence, the reliability of intrusion detection dataset is


$$\begin{array}{c} \Omega_{A}=\varpi_{I}S_{I}+\varpi_{A}S_{A}+\varpi_{C}S_{C}+\varpi_{D}S_{D}+\varpi_{B}S_{B}\\ \;=0\mathrm{.087}*97.41\%+0\mathrm{.172}*99.92\%+0\mathrm{.358}*99.98\%+0\mathrm{.363}*90.18\%+0\mathrm{.02}*41.05\%\\ \;=95\mathrm{.01}\% \end{array}$$

**Fig 4:**
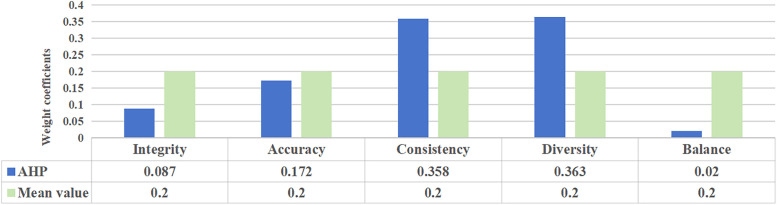
The weight coefficients of evaluation indicators.

The evaluation score of CICIDS2017 dataset is 95.01%, by using the AHP. The distribution of weight coefficients for the two methods is shown in Fig 4. It can be seen that, the AHP can more accurately identify core indicators such as accuracy, consistency and diversity, by assign them higher weights, which makes the final evaluation results more in line with the actual data requirements situation. In contrast, the integrity and balance indicators are given lower weights. The proportion of normal samples in the overall sample is quite large. Therefore, the impact of data integrity on the overall contribution is weakened accordingly. Meanwhile, the imbalance of intrusion detection data is objective and difficult to change due to the different complexity of different attack types.

### B.The evaluation for intrusion detection data in a AI-enabled IDS

**Fig 5:**
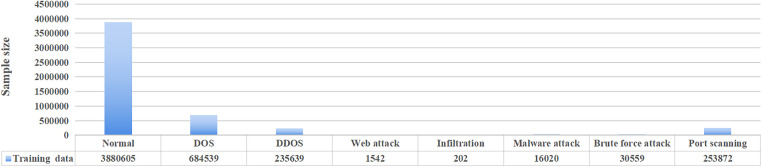
The distribution of intrusion detection data type.

In this example, the practical evaluation, focusing on an AI-enabled IDS developed and trained by a company in Hebei Province, China. In this evaluated dataset, there are 72 input features, including various aspects of network traffic, such as the basic information of the network connection (source IP, destination IP, source port, destination port, etc.), the timestamps of the packets, the protocol type, the size of the traffic, etc. These features can reflect the basic attributes of network connections, and abnormal network behaviors can be detected by analyzing these attributes. In terms of data volume, the training data totaled 5,102,978 items and the common types in intrusion detection scenarios are shown in Fig 5, which covers 7 major attack types such as DOS attacks, DDOS attacks, and Web attacks. Based on the actual situation of these data, a comprehensive assessment of the system’s reliability will be carried out in-depth, providing solid data support and decision-making basis for the optimization and improvement of the system.

### B.1 The indicators evaluation for intrusion detection

**Fig 6:**
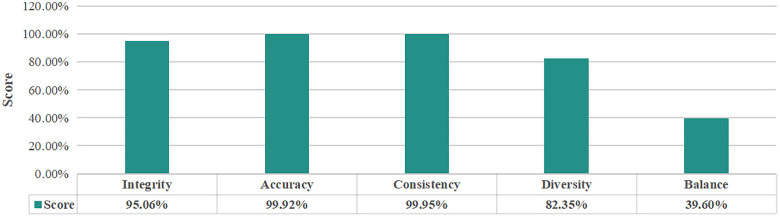
The evaluation results of indicators for intrusion detection data.

The five key indicators are also used to evaluation the reliability of this intrusion detection data and the results are summarized in the Fig 6. It is worth noting that timeliness is important for assessing data reliability during application. However, based on both the publicly benchmark data and actual data, this evaluation is limited to the training stage of AI models. Therefore, we will not consider the timeliness of data transmission for the time being.

**Integrity:** Firstly, null value detection is carried out and 2355 samples containing NaN and null value are detected, so the null value detection score is 99.95%. Moreover, the data mutation detection score, by calculating the root mean square of the current sample 621.46, and calculating the average root mean square value of 10 historical samples of the same batch is 689.3, is 90.16%. The overall score of integrity is 95.06%.

**Accuracy:** A total of 4026 samples were found to contain outliers by 3Sigma principle, so the accuracy score is 99.92%.

**Consistency:** The consistency of format was detected first, and 0 items are found. In the consistency of content detection, 216 items are found to have label inconsistency and in the consistency of relationship detection, 6,268 logical relationships are found to have abnormal data. According to the given calculation method in this paper, the overall score of consistency is 99.95%.

**Diversity:** In this intrusion detection dataset of this evaluation, there are 8 types of labels (1 normal + 7 attack types). The distribution probabilities are 0.76, 0.13, 0.05, 0.0003, 0.00004, 0.003, 0.006, 0.497 separately. Then, Shannon entropy calculation is performed: 0.208, 0.265, 0.1498, 0.0024, 0.0004, 0.0174, 0.0307, 0.1498, and the score of diversity is 82.35%.

**Balance:** The value of PEI is 0.396, thus the score of balance is 39.6%.

### B.2 Comprehensive evaluation for intrusion detection

After evaluating scores of the above indicators, the comprehensive evaluation will be given. Since the data used in this scenario is consistent with the benchmark dataset in terms of collection type, label type, and application scenario, the original weights by AHP can be directly applied to uniformly evaluate the completeness, accuracy, consistency, diversity and balance of the data. The comprehensive evaluation result is


$$\begin{array}{c} \begin{array}{c} \Omega_{B}\;=0\mathrm{.087}*95.06\%+0\mathrm{.172}*99.92\%+0\mathrm{.358}*99.95\%+0\mathrm{.363}*82.35\%+0\mathrm{.02}*39.6\%\\ \;\;\;=91\mathrm{.96}\% \end{array} \end{array}$$

The reliability score of the data is 91.96%. Furthermore, focusing on the core indicators, it can provide clearer guidance on data reliability improvement for researchers. Following a series of data processing operations, including deduplication, sample augmentation and denoising from this intrusion detection dataset, the data quality is improved. For instance, for the duplicate samples, after searching, duplicate samples are directly deleted. It may increase the imbalance among sample categories. Thus, to mitigate the bias degree of samples, sample augmentation technology was adopted. For handling imbalanced datasets, SMOTE (Synthetic Minority Over-sampling Technique) can increase the number of samples in the minority class by creating synthetic samples rather than simply duplicating existing ones, which enhances the model’s ability to recognize the minority categories. Therefore, SMOTE was used to generate samples to expand the minority of attack-type (Web attack, Infiltration, Malware attack, Brute force attack). However, the data, consisting of the raw and new samples, may contain the noise. Thus, the 3-sigma was used for detecting noise again and eliminate abnormal samples. Next, we conducted a comprehensive reassessment of the dataset, yielding a score of 94.6%, marking a 2.64% improvement over the original sample’s score. It is worth noting that it is one-sided to measure the reliability of the AI-enable IDS by focusing only on the increase in comprehensive scores. Therefore, to empirically test the specific impact of data preprocessing on AI model performance, the stacked denoising encoders (SDA) method in this IDS is employed for experimentation. The SDA are trained with the raw dataset (SDA-R) and the modified dataset (SDA-M), respectively. Notably, this training dataset exhibits a highly imbalanced distribution of positive and negative samples. Consequently, to provide a more intuitive perspective on the model’s ability to recognize minority classes, when dealing with imbalanced datasets, the precision, recall rate and F1-score are adopted


{QPre=τi/τiσi\nulldelimiterspaceσiQRe\nolimitsc=τi/τiTi\nulldelimiterspaceTiQF=2QPre*QRe\nolimitsc/2QPre*QRe\nolimitsc(QPre+QRe\nolimitsc)\nulldelimiterspace(QPre+QRe\nolimitsc), (15)

where *τ*_*i*_ represents the actual number of *i*th label type, *σ*_*i*_ is the number of *i*th label type predicted by the SDA and *T*_*i*_ is the sample size of the *i*th label type. Furthermore, the experimental results are compared with those of SVM-ELM-MK [24], DA-LGBM [25], SAE, GAN-rfc [27], ISSAE [10] and MCNN [12] with the significance test *p*-value.

The precision and recall rate comparisons of different AI models are displayed in Table 5 with the significance test p < 0.05.

**Table 5:** The precision and recall of different AI models with p < 0.05.

Type (%)	Normal		DoS		DDoS		Web Attack		Infiltration		Malware attack		Brute force attack		PortScan	
	Q_Pre_	Q_Rec_	Q_Pre_	Q_Rec_	Q_Pre_	Q_Rec_	Q_Pre_	Q_Rec_	Q_Pre_	Q_Rec_	Q_Pre_	Q_Rec_	Q_Pre_	Q_Rec_	Q_Pre_	Q_Rec_
**SDA-M**	**93.14**	**96.58**	**95.48**	**97.56**	**97.16**	**99.35**	**95.98**	**93.85**	**96.38**	**92.24**	**97.87**	**99.33**	**89.22**	**92.08**	**95.86**	**99.22**
SDA-R	83.63	87.77	87.62	91.28	85.21	86.46	91.63	89.7	96.15	95.11	83.98	86.44	92.23	95.09	91.46	95.82
SVM-ELM-MK [24]	80.82	86.76	88.78	90.36	83.5	85.48	93.36	88.18	86.87	85.33	83.16	85.46	83.49	86.35	89.12	90.48
DA-LGBM [25]	84.61	88.12	86.62	92.94	88.16	89.4	88.73	72.6	88.14	84.2	78.25	80.61	74.27	78.13	80.24	82.6
SAE	88.70	92.03	89.03	90.29	95.53	97.72	92.42	90.29	89.39	90.15	94.74	97.2	80.86	84.72	94.27	96.63
GAN-rfc [27]	95.16	99.86	89.26	90.68	97.18	98.98	95.01	91.98	84.22	74.98	97.52	98.96	70.1	74.96	96.62	98.98
ISSAE [10]	92.39	95.29	91.66	94.08	97.96	99.68	93.61	85.98	99.39	98.98	96.17	98.51	95.1	98.96	95.62	97.98
MCNN [12]	95.28	98.94	95.42	97.11	98.05	99.42	95.82	92.78	95.69	91.65	96.04	99.4	86.77	91.63	97.61	99.97

**Fig 7:**
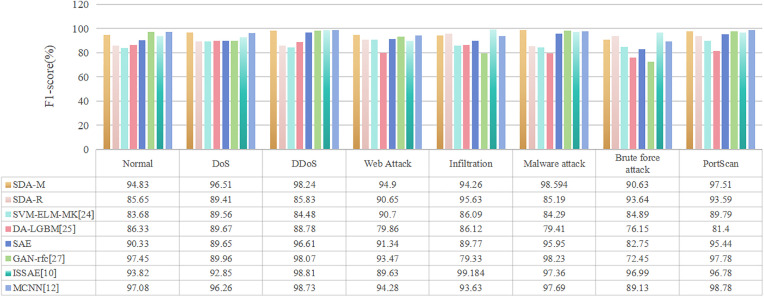
The comparisons of F1-score of different AI models with *p* < 0.05.

It can be seen that SDA-M improves the precision and recall rate across attack categories, comparing with SDA-R. Moreover, the experimental results of SDA-M significantly outperform that of the well-designed SVM-ELM-MK and the DA-LGBM. Moreover, the performances of SDA-N demonstrates comparable competitiveness when compared to some of the modified deep learning algorithms, such as GAN-rfc, ISSAE, and MCNN. Furthermore, as shown in Table 5, the model exhibits high precision but low recall in the minority class. Conversely, in the majority class, the model demonstrates low precision but high recall. Therefore, to effectively evaluate model performance, the F1 score is adopted, with results presented in Fig 7. the F1 score of SDA-M still better than SDA-R in all categories. Consequently, based on the above analysis, by comprehensively assessing and enhancing the intrusion detection dataset, we can provide AI models with abundant, accurate, and diverse samples. This enables the AI models to learn more nuanced details and variations, thereby reducing the occurrence of errors and misleading results, ultimately strengthening the reliability of IDS. Additionally, the proposed MICEM can not only perform offline evaluations, such as assessing data before model retraining, but also evaluate the latest traffic data through sliding windows (e.g., every 5–30 minutes). It is worth noting that in the online evaluation scene, the evaluation frequency of MICEM must balance real-time responsiveness with comprehensive coverage. A hybrid strategy, combining a base cycle with dynamic triggers, is recommended, with computational overhead managed through sampling, distributed computing, and hardware acceleration

## IV. Conclusion

In this paper, a MICEM is proposed to estimate the data reliability for a practical AI-enabled IDS from the data perspective. In this evaluation process, multiple crucial indicators are designed to evaluate intrusion detection data condition separately, which provides a clear warning of the potential risks in the training data. Then, a comprehensive evaluation method, incorporating the AHP, to evaluate the quality of the entire intrusion detection data, thus guaranteeing the usability and reliability of AI-Enabled IDS. Finally, by evaluating the reliability of intrusion detection data in real application scenarios, the effectiveness and practicality of the proposed method are fully verified. It is worth further clarifying that the proposed MICEM can also applicable to other similar scenarios, by the detailed description of calculation steps in this paper. In our future research, more indicators and sub-items will be designed and introduced for the AI model in IDS with a safe and efficient manner.
